# Brazilian dentists’ knowledge, skills, and attitudes towards minimal intervention dentistry: development and psychometric analysis

**DOI:** 10.1590/1807-3107bor-2026.vol40.039

**Published:** 2026-06-22

**Authors:** Regina Cardoso de Moura, Matheus França Perazzo, Soraya Coelho Leal, Lucianne Cople Maia, Carla Massignan

**Affiliations:** (a) Universidade de Brasília – UnB, School of Health Sciences, Department of Dentistry, Brasília, DF, Brazil.; (b) Universidade Federal de Goiás – UFGO, School of Dentistry, Department of Social Dentistry, Goiânia, GO, Brazil.; (c) Universidade Federal do Rio de Janeiro – UFRJ, Schoool of Dentistry, Department of Pediatric Dentistry and Orthodontics, Rio de Janeiro, RJ, Brazil.

**Keywords:** Validation Study, Psychometrics, Evidence-Based Dentistry, Dental Caries, Health Knowledge, Attitudes, Practice

## Abstract

To date, no instrument with demonstrated structural validity has been available to assess dentists’ knowledge, skills, and attitudes regarding the core concepts of Minimal Intervention Dentistry (MID). Therefore, this study aimed to develop and evaluate the psychometric properties of a self-administered instrument designed to measure Brazilian dentists’ knowledge, skills, and attitudes towards MID. A cross-sectional study was conducted among dentists practicing in the Federal District of Brazil to evaluate the psychometric properties of the instrument. Item generation was followed by expert review by Brazilian MID specialists. Subsequently, two focus groups and a pre-test were conducted to assess item wording, sequence, and comprehensibility. After the necessary revisions, the instrument was administered to 454 dentists. Exploratory Factor Analysis, combined with parallel analysis, was performed (p < 0.05). A total of 404 dentists comprised the final sample for the psychometric analyses. Parallel analysis indicated a two-factor solution as the most representative, corresponding to knowledge, skills, and attitudes. Composite reliability was adequate for all factors (≥ 0.40). The factorial structure showed acceptable model fit indices, except for the RMSEA (RMSEA = 0.092). Unidimensionality was rejected, as UniCo = 0.939 (0.907–0.975) and ECV = 0.762 (0.719–0.830) did not reach the recommended thresholds. The findings suggest that the instrument presents an appropriate structure and satisfactory reliability for measuring dentists’ knowledge, skills, and attitudes related to MID. This is the first study in the field of MID competencies to comprehensively report the steps involved in constructing, adapting, and psychometrically evaluating an instrument for dentists.

## Introduction

The development of a measurement instrument with adequate psychometric properties requires a systematic and sequential process.^1^ The first stage involves item development, which is directly related to content validity and includes a literature review, complementary procedures such as interviews and expert consultation, operational definition of constructs, and item formulation.^1^ This stage results in a preliminary version of the instrument. The second stage concerns data collection and includes the application of the preliminary instrument to focus groups, a pilot sample, and the target population.^1^ The third stage comprises statistical analyses, evaluation of measurement properties, and the refinement and finalization of the instrument.^1^

Minimal Intervention Dentistry (MID) can be defined as a holistic, team-based approach aimed at maintaining long-term oral health through preventive, patient-centered, and behavior-oriented care plans, combined with the conscientious management of patients’ needs, preferences, and expectations.^2^ However, translating MID-related knowledge into routine clinical practice remains challenging, partly due to the extensive and heterogeneous body of information available on the subject.^3^

Evidence of the gap between MID knowledge and clinical practice is illustrated by findings from systematic reviews. One review demonstrated that in nearly half of the included studies, dentists failed to adopt evidence-based strategies for the management of deep carious lesions in permanent teeth.^3^ Another review showed that although dentists generally exhibit adequate knowledge of MID principles, their attitudes and clinical practices remain limited. This review reported stronger theoretical understanding—particularly regarding caries etiology, diagnosis, risk assessment, and fluoride use—alongside lower adherence to conservative clinical approaches such as restoration repair and Atraumatic Restorative Treatment (ART). Additionally, methodological heterogeneity and the lack of validated instruments were highlighted, reinforcing the need for standardized tools to assess dentists’ knowledge and practices related to MID.^4^ These gaps justify the development of a valid and reliable measurement instrument.

In the present study, competencies were conceptualized as the integration of knowledge, skills, and attitudes, based on Zarifian's framework, which defines competencies across three dimensions: knowledge (knowing), skills (know-how), and attitudes (willingness to act).^5^ The competency-related items were organized into thematic areas aligned with the four core concepts of MID proposed by Walsh: recognition, reduction, regeneration, and repair.^6^ To the best of the authors’ knowledge, no instrument with established structural validity has been developed to assess dentists’ competencies—knowledge, skills, and attitudes—related to the fundamental principles of MID. Thus, this study aims to develop and evaluate the psychometric properties of a new instrument designed to measure Brazilian dentists’ knowledge, skills, and attitudes towards MID.

## Methods

### Population, context, and collection period

This study is reported in accordance with the COSMIN reporting guideline for studies on measurement properties of patient-reported outcome measures.^7^

A cross-sectional study was conducted to validate an instrument developed to assess knowledge, skills, and attitudes related to Minimal Intervention Dentistry (MID). The self-administered questionnaire was designed using the Google Forms platform, and data were collected online. Eligible participants were dentists practicing in the Federal District (FD), Brazil, at the time of the study. Dentists who were not registered with the Regional Council of Dentistry of the Federal District were excluded.

Non-probabilistic snowball sampling was adopted because it was not feasible to contact all members of the target population to obtain a random sample.^8,9^ The instrument was distributed via URL and QR code through email, social media (group and individual accounts), and printed flyers distributed in dental clinics throughout the FD. Participants were encouraged to invite one or more dentists from their professional networks to participate.

Data collection was carried out between November 2021 and March 2023. The study complied with Resolution No. 466/2012 of the Brazilian National Health Council and was approved by the Research Ethics Committee of the Faculty of Health Sciences of the University of Brasília (CAAE: 47639021.8.0000.0030). It was conducted following the resolution 466/2012 of the Brazilian National Health Council.

### Development, adaptation and psychometric evidence assessment of the instrument

The development of the instrument was based on previous studies addressing competencies in MID^10^ and on available scientific evidence related to MID.^6,11,12^ The proposed instrument comprised seven sections, described in the supplementary material (https://l1nq.com/L98u7). Sections 1 to 4 included the informed consent form, eligibility questions, and items assessing participants’ sociodemographic characteristics, educational background, and previous experience with MID. Sections 5 to 7 contained items designed to assess dentists’ knowledge, skills, and attitudes related to MID, all answered using a five-point Likert scale. Additional sections included two Likert-scale items assessing perceived barriers to MID knowledge and practice, as well as one open-ended item allowing participants to provide additional comments or suggestions.

Initially, the instrument was evaluated by a panel of Brazilian experts with recognized scientific production in MID between 1996 and 2021. Potential experts were identified through a SCOPUS database search conducted on September 28, 2021 (n = 168), using the following Health Sciences Descriptors (DeCS/MeSH): "Minimal Intervention Dentistry" AND "Dentists" AND "Health Knowledge, Attitudes, Practice," along with related terms (Figure 1). The experts were invited via email to voluntarily and anonymously assess the technical-scientific content, wording, and clarity of the items. Twelve experts agreed to participate.

**Figure:**
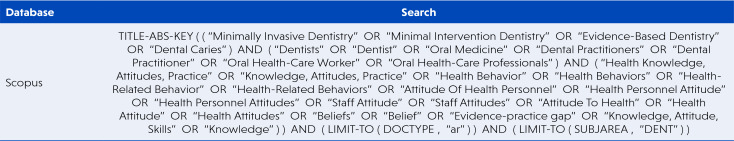
Database search strategy.

Each item was rated for relevance on a four-point Likert scale (1 = not relevant; 2 = slightly relevant; 3 = relevant; 4 = very relevant), with an additional field for qualitative suggestions. Items that received scores of 1 or 2 from at least 10% of the experts were reformulated based on the experts’ suggestions until consensus was reached among the research team.^13^

Following expert review and revision, the instrument was evaluated in two focus groups to assess comprehension by the target population. The focus groups comprised three and two dentists, respectively, selected by convenience sampling from different dental specialties and who did not participate in other stages of the study. Meetings were conducted online, with the instrument sent to participants in advance and displayed during the sessions. All items were read aloud, and participants were asked to comment on their understanding and to suggest improvements. All feedback was documented, and consensual revisions were subsequently incorporated to enhance clarity and readability.

Prior to the main data collection, the instrument was pre-tested with 30 dentists acting as judge-evaluators to assess clarity, adequacy, and comprehensibility of the items. These dentists, colleagues of the authors from different Brazilian states, were recruited via social media using convenience sampling. At this stage, the Content Validity Coefficient (CVC) was calculated to evaluate item suitability.^14^ Each judge-evaluator assessed the items based on clarity, adequacy, and comprehension using a five-point Likert scale ranging from 1 ("strongly disagree") to 5 ("strongly agree"). Items with a CVC greater than 0.80 were considered acceptable.^14,15^ All items met this criterion, with an overall CVC of 0.93. Responses from this stage were not included in the final analyses.

### Measurement

#### Knowledge, skills, and attitudes related to MID

This section comprised 17 structured items answered on a five-point Likert scale, designed to assess dentists’ knowledge, skills, and attitudes related to MID (Table 1). Total scores ranged from 17 to 85, with higher scores indicating greater competency in the assessed domains.

**Table 1:** MID Knowledge, Skills, and Attitudes Instrument.

Thematic areas	Competencies assessed		
	Knowledge	Skill	Attitude
MID (General)	**Item 1.** How much do you know about Minimal Intervention Dentistry in the management of dental caries?	**Item 2.** To what extent do you agree with the following statement: The goal of Minimal Intervention Dentistry is to maintain teeth healthy and functional throughout life, and it involves implementing key strategies to prevent caries lesions, including early caries detection and risk assessment; remineralization of demineralized enamel and dentin; optimal caries prevention measures; minimally invasive operative interventions; and repair rather than replacement of restorations.	**Item 3.** How often do you apply Minimal Intervention Dentistry in the management of dental caries in your daily clinical practice??
**Recognition, reduction, and remineralization**	**Item 4.** How much do you know about identifying caries risk factors?	**Item 7.** To what extent do you agree with the following statement: Assessing the frequency of consumption of sugar-containing foods is a good indicator of future caries risk.	**Item 10.** How often do you assess your patients' risk of dental caries?
	**Item 5.** How much do you know about controlling the onset of dental caries?	**Item 8.** To what extent do you agree with the following statement: Exposure to fluoride sources is a good indicator of protection against dental caries.	**Item 11.** How often do you advise patients at high risk for dental caries to reduce the consumption of sugar-containing foods?
	**Item 6.** How much do you know about controlling the progression of dental caries?	**Item 9.** To what extent do you agree with the following statement: In patients at high risk for dental caries, behavior changes, such as reducing the frequency of consumption of sugar-containing foods, are an important factor in controlling disease onset.	**Item 12.** How often do you recommend daily toothbrushing with fluoridated toothpaste for patients at high risk for dental caries?
**Minimally invasive restorations and restoration repair**	**Item 13.** How much do you know about minimally invasive restorative procedures?	**Item 14.** To what extent do you agree with the following statement: The removal of carious dentin should vary according to lesion depth and be more conservative in deep lesions to avoid pulp exposure in vital teeth.	**Item 16.** How often do you intentionally leave soft carious tissue on the pulpal floor to avoid pulp exposure when restoring deep carious lesions in vital teeth?
		**Item 15.** To what extent do you agree with the following statement: In defective restorations, repair should be considered before opting for complete removal and replacement	**Item 17.** How often do you perform restoration repair rather than complete replacement when managing defective restorations?

* MID: minimal intervention dentistry.

#### Barriers to the MID

This section consisted of two structured items answered on a five-point Likert scale to assess perceived barriers to MID knowledge and practice. Scores ranged from 2 to 10, with higher scores indicating greater perceived difficulty

### Sociodemographic characteristics, professional profile, and experience with MID

This section collected information on gender, age, region of residence, type of undergraduate institution (public or private), years of professional experience, highest academic degree, postgraduate specialty, time since completion of the most recent postgraduate degree, main professional activity, previous training in MID, training setting, and sources of information on MID.

### Data analysis plan

Data processing and statistical analyses were performed using SPSS (version 23) and Factor (version 11.05). Exploratory Factor Analysis (EFA) was conducted to evaluate the factorial structure of the knowledge, skills, and attitudes scale related to MID. Analyses were based on a polychoric correlation matrix using the Robust Diagonally Weighted Least Squares (RDWLS) extraction method.^16^ The number of factors to be retained was determined using parallel analysis with random permutation of the data ^17^ followed by Robust Promin rotation.^18^

Composite reliability was calculated based on standardized factor loadings and error variances.^19^ Unidimensionality was assessed using the following indices: Unidimensional Congruence (UniCo > 0.95), Explained Common Variance (ECV > 0.80), and Mean of Item Residual Absolute Loadings (MIREAL < 0.30). Model fit was evaluated using the Root Mean Square Error of Approximation (RMSEA), Comparative Fit Index (CFI), and Tucker–Lewis Index (TLI).^10^ Acceptable model fit was defined as RMSEA < 0.06, CFI > 0.90, and TLI > 0.90.^20^

## Results

The two focus groups comprised three and two dentists, respectively, all of whom were female, with a mean age of 44 years. All five participants held a specialization as their highest academic degree, and some had completed more than one specialty. The areas of specialization included orthodontics (12.5%), general dentistry (12.5%), prosthodontics (25.0%), implantology (12.5%), occupational dentistry (12.5%), pediatric dentistry (12.5%), and endodontics (12.5%).

In the pre-test phase, 30 dentists participated, of whom 73.3% (n = 22) were female, 23.2% (n = 7) were male, and one participant preferred not to disclose their gender. Additional details are presented in Table 2.

**Table 2:** Sociodemographic characteristics, education, and professional experience of dentists participating in the pre-test, Brazil, 2023

Variables		
1. Gender (n = 30).		n (%)
	Female	22 (73.3)
	Male	7 (23.3)
	Prefer not to disclose	1 (3.3)
2. Age in years (n = 30).		mean (± SD)
		36.6 (± 7.1)
3. Years of professional experience (n = 30).		mean (± SD)
		12.3 (±7.6)
4. Professional activity (n = 30)		n (%)
	Pediatric dentistry	8 (26.7)
	Orthodontics	7 (23.3)
	Prosthodontics	6 (20)
	Dentistry	3 (10)
	Temporomandibular disorder and orofacial pain	3 (10)
	Orofacial harmonization	3 (10)
	Endodontics	2 (6.7)
	Geriatric dentistry	2 (6.7)
	Dentofacial orthopedics:	2 (6.7)
	Implantology	1 (3.3)
	Public health dentistry	1 (3.3)
	Dentistry for patients with special needs	1 (3.3)
	Dental radiology	1 (3.3)
	Other postgraduate areas	3 (10)
	No postgraduate degree	3 (10)
5. Highest academic degree		n (%)
	Bachelor's degree	4 (13.3)
	Specialized degree	16 (53,3)
	Master's degree	6 (20.0)
	Doctoral degree	4 (13.3)

Of the 454 dentists who accessed the questionnaire, professionals who were not eligible for the study (n = 20; 4.4%) or who did not consent to participate (n = 30; 6.6%) were excluded. Thus, the final sample for the psychometric analyses of the MID knowledge, skills, and attitudes scale consisted of 404 dentists. Among these participants, the majority were female (74.0%), worked in their own or shared private practices (66.6%), and reported a specialization as their highest academic degree (61.1%).

Bartlett's test of sphericity (χ^2^ = 2481.7; df = 120; p < 0.01) and the Kaiser–Meyer–Olkin (KMO) measure of sampling adequacy (0.83) indicated that the item correlation matrix was suitable for factor analysis. Parallel analysis suggested a two-factor solution as the most representative of the data, as these two factors from the observed data explained a greater proportion of variance than the corresponding factors derived from random data.

In the preliminary analysis, item 3 ("How often do you apply Minimal Intervention Dentistry in the management of dental caries in your daily clinical practice?") exhibited a cross-loading pattern, with factor loadings of 0.383 and 0.325 on Factors 1 and 2, respectively. Consequently, this item was removed, and a new analysis was conducted. The two-factor structure was retained and reasonably reflected the theoretical division of the items, with Factor 1 (F1) corresponding to the Knowledge dimension and Factor 2 (F2) to the Skills and Attitudes dimension. Only item 10 ("How often do you assess your patient's caries risk?") did not load onto the theoretically expected Skills and Attitudes dimension. Factor loadings and composite reliability indices are presented in Table 3.

**Table 3:** Final factorial structure of competencies related to MID.

Items	Factor 1	Factor 2
	Knowledge in MID	Skills and attitudes in MID
Item 1	**0.498**	0.285
Item 2	-0.002	**0.626**
Item 4	**0.709**	-0.104
Item 5	**0.880**	-0.045
Item 6	**1.008**	-0.162
Item 7	-0.108	**0.589**
Item 8	0.026	**0.377**
Item 9	-0.050	**0.598**
Item 10	**0.456**	0.078
Item 11	0.007	**0.499**
Item 12	0.141	**0.395**
Item 13	**0.599**	0.205
Item 14	0.110	**0.452**
Item 15	-0.099	**0.718**
Item 16	0.154	**0.403**
Item 17	-0.023	**0.714**
Composite reliability	0.856	0.806

* MID: minimal intervention dentistry.

Most items demonstrated adequate to high factor loadings on their respective factors, and no cross-loading patterns were observed in the second analysis. Composite reliability values were acceptable for both factors, exceeding the minimum threshold of 0.40. The factorial structure demonstrated acceptable fit indices (χ^2^ = 235.09; df = 89; p > 0.001; CFI = 0.934; TLI = 0.910), with the exception of the RMSEA (RMSEA = 0.092), which indicated a mediocre fit. Unidimensionality was rejected, as the UniCo value (0.939; 95% CI: 0.907–0.975) and the ECV value (0.762; 95% CI: 0.719–0.830) did not reach the recommended thresholds, whereas only the MIREAL value (0.288; 95% CI: 0.254–0.323) met the criterion.

## Discussion

The present study aimed to develop and evaluate the psychometric properties of a new instrument designed to measure Brazilian dentists’ knowledge, skills, and attitudes related to Minimal Intervention Dentistry (MID). This study reports the results of the instrument development process and the exploratory factor analyses performed. The instrument demonstrated satisfactory performance in the Exploratory Factor Analysis, and the findings provide psychometric evidence supporting its use.

This study has certain limitations. Its cross-sectional design may not fully reflect the complexity of dentists’ competencies and may be subject to recall bias. To minimize these limitations, several methodological steps were undertaken, including systematic item development, evaluation by a panel of Brazilian MID experts, focus group discussions with the target population, and a pre-test to assess clarity and content validity. Another limitation concerns the use of non-probabilistic sampling, which may affect sample representativeness; however, this was mitigated through careful participant selection at the outset of the study. As the study was conducted within a specific local context, the findings and their interpretation should not be generalized to other populations without further validation.

Unidimensionality—defined by Hu and Bentler (1999) as the condition in which a single latent construct explains item covariances—was rejected. Establishing unidimensionality is important for ensuring a coherent and interpretable measurement model, as it confirms that item covariances are primarily accounted for by a single latent variable rather than by multiple unrelated factors.^20^ In the present study, the internal structure was found to be multidimensional, comprising a two-factor model that was consistent with the theoretical framework adopted by the researchers. It is noteworthy that multidimensionality is inherent to competency-based assessments,^4^ which supports both the conceptual model proposed and the factorial solution obtained through exploratory factor analysis.

The factor loadings obtained were positive and exceeded the minimum acceptable threshold of 0.30, although acceptable values may vary depending on the type of study.^21^ Most items showed adequate alignment with their respective factors. Item 3 ("How often do you apply Minimal Intervention Dentistry in the management of dental caries in your daily clinical practice?") exhibited factor loadings above 0.30 on both factors and was therefore excluded from the final model. After its removal, item 10 ("How often do you assess your patient's risk of dental caries?") did not load onto the theoretically expected Skills and Attitudes dimension. These findings indicate the need for further analyses in future studies with different samples to confirm the factorial structure and to strengthen the evidence of construct validity.

With regard to composite reliability, the values obtained for both factors were adequate. Composite reliability was chosen as the reliability estimator because it avoids the underestimation commonly associated with Cronbach's alpha.^19^

Regarding the originality of this study, previous research addressing dentists’ competencies in MID has been published.^10,22–24^ However, three of these studies reported only pre-test and pilot phases prior to instrument application,^10,22,23^ and only one study reported pre-testing, adaptation, and validation procedures.^24^ Nevertheless, the methodological steps in that study were not clearly described.^24^ In contrast, the present study is the first in the field of MID competencies to comprehensively report all stages of instrument construction, adaptation, and psychometric evaluation for dentists.

A general trend toward positive responses to the assessed items was observed. Replication of this study in different contexts and populations is recommended to verify whether the factorial solution remains stable and to further strengthen the validity evidence for the instrument.

## Conclusion

The instrument demonstrated satisfactory performance in the Exploratory Factor Analysis, and the findings provide psychometric evidence supporting its use for assessing dentists’ knowledge, skills, and attitudes related to Minimal Intervention Dentistry. This is the first study in the field of MID competencies to comprehensively report the development, adaptation, and psychometric evaluation of an instrument for dentists. Nevertheless, further studies are recommended to expand and strengthen the evidence of its validity.

## Data Availability

The authors declare that all data generated or analyzed during this study are included in this published article.
